# 

**Published:** 1887-04

**Authors:** 


### TABLE OF CONTENTS.

ORIGINAL AND SELECTED ARTICLES.

Lawson Tait, F. R. C. S. .... 133
Nævus .... 137
Antipyrin, by H. E. Stroud, of Colorado .... 138
Convulsions .... 139
Case of Laparotomy, by J. G. Earnest, M. D., of Atlanta, Ga .... 140
A Twinchild with Hydrocephalus, Requiring the use of Perforator and Hook for Delivery, by J. McF Gaston, M.D .... 141
Pelvic Inflammation, by W. Ewing, M. D .... 142
New York Academy of Medicine .... 146
A Case of Epilepsy Apparently Due to Genital Irritation, and Cured by Circumcision, by Wharton Sinkler, M.D .... 147
Salicylic Acid; How does it Cure Rheumatism 148

ABSTRACTS AND GLEANINGS.

Rest for Painful Eyes Not Always Effectual... 149
Salicylate of Ammonium .... 152
Anæsthetics .... 152
A Suture that is Painless and Leaves No Scar 154
The Treatment of Renal Calculus; the Treatment of Compound Fractures .... 155
The Removal of Superfluous Hair .... 156
Surgical Infection; Antipyrine in Pneumonia 157
The Influence of Antipyrine on the Temperature and Tisue Change in Children .... 158
Embalming; Antipyrin in Typhoid Fever .... 159
Strangulated Hernia Treated by Puncture; Before attemping to remove foreign bodies 160
Perchloride of Iron in Anthrax; The Proper Use of Antipyrine; "Black Eye." .... 161
Fracture of the Patella; Hellers's Albumin Test; Treatment of Ingrowing Toe-nail; Rival to Cocaine; Bi-Tartrate of Potash in Pregnancy .... 162

SCIENTIFIC ITEMS.

Insects; Circulation Not Essential to Life; Snow .... 163
Carboniferous Age; A New Anti-Rabies Inoculation; Hermit Crab .... 164

PRACTICAL NOTES AND FORMULÆ.

Remedy for Whooping Cough; For Acute Bronchitis; Hypodermic Medication in Severe Uterine Hemorrhage; Inflammation of the Hair Follicles within the Nares .... 165
T. A. Edison's Anæsthetic; For Cancer of the Stomach; Camphorated Dover's; Terbene... 166
Pills for Phthisis; Treatment of Syphilis; For Hysteria; For Chronic Bronchitis; Dr. Rorick's Formula for Injecting Hemorrhoids... 167

EDITORIALS AND MISCELLANEOUS.

Editorial Brevities .... 168
Commencement Exercises of the Southern Medical College .... 169
The American Medical Association; The Medical Association of Georgia; Liberality of Parke, Davis & Co .... 174
Books and Pamphlets Received .... 175
Receipted .... 176

